# Характеристики закупок сахароснижающих лекарственных средств в коммерческом секторе в динамике за 2016–2020 гг.

**DOI:** 10.14341/probl13200

**Published:** 2023-08-30

**Authors:** Д. В. Куркин, Е. В. Макарова, И. С. Крысанов, Д. А. Бакулин, А. И. Робертус, О. В. Иванова, Ю. А. Колосов, Р. А. Кудрин

**Affiliations:** Московский государственный медицинский стоматологический университет им. А.И. Евдокимова; Московский государственный медицинский стоматологический университет им. А.И. Евдокимова; Университет Сантьяго де Компостела; Московский государственный медицинский стоматологический университет им. А.И. Евдокимова; ФГБОУ ВО «РОСБИОТЕХ»; Волгоградский государственный медицинский университет; Московский государственный медицинский стоматологический университет им. А.И. Евдокимова; Российский научно-исследовательский медицинский университет им. Н.И. Пирогова; Московский государственный медицинский стоматологический университет им. А.И. Евдокимова; Московский государственный медицинский стоматологический университет им. А.И. Евдокимова; Волгоградский государственный медицинский университет

**Keywords:** сахарный диабет 2 типа, оборот лекарственных средств, иДПП4, арГПП1, иНГЛТ2, сульфонилмочевина, метформин, рынок сахароснижающих средств

## Abstract

**ОБОСНОВАНИЕ:**

ОБОСНОВАНИЕ. Закупки лекарственных средств (ЛС) отражают востребованность и частоту назначения тех или иных препаратов, что позволяет оценить качество оказания медицинской помощи и соблюдение стандартов. Российский фармацевтический рынок динамично развивается и расширяется, следовательно, коммерческий сектор оборота лекарств является его значительной частью и должен быть исследован наравне с государственными закупками. Учитывая значительное число пациентов с диагнозом сахарного диабета (СД) в нашей стране, мы сочли целесообразным и интересным проанализировать структуру и объем оборота противодиабетических ЛС в розничной торговле за 5 лет.ЦЕЛЬ. Оценить динамику стоимости и объемов реализации сахароснижающих препаратов в коммерческом секторе за 2019–2020 гг. в сравнении с 2016 г.МАТЕРИАЛЫ И МЕТОДЫ. Был проведен анализ данных по закупкам сахароснижающих препаратов в аптечные организации за 2016 и 2019–2020 гг. по данным 95 257 аптек.РЕЗУЛЬТАТЫ.В 2020 г., в сравнении с 2016-м, отмечается значимый рост закупок в количестве упаковок (+14 952 897 шт.) и общей сумме закупок (+9 377 975 722 руб.) на фоне увеличения средневзвешенной цены на одну коробку препарата на 199,57 руб. Снизилась средняя цена на ингибиторы дипептидилпептидазы-4 (иДПП4). Стоимость упаковки метформина остается одной из самых низких, уступая только глибенкламиду и гликлазиду. К самым дорогим препаратам относятся все агонисты рецепторов глюкагоноподобного пептида типа 1 (арГПП1). В 2 раза снизились закупки инсулинов, в 10 раз возросли затраты на арГПП1, в 9,5 раза — на ингибиторы натрий-глюкозного транспортера типа 2, в 2,1 раза — на иДПП4. В 2020 г. лидерами по количеству закупленных упаковок остаются метформин, гликлазид, комбинация глибенкламида с метформином, глибенкламид и вилдаглиптин. Лидерами закупок по доле в бюджете являются метформин, гликлазид, лираглутид, вилдаглиптин и дапаглифлозин.ЗАКЛЮЧЕНИЕ. Отмечаются позитивные тенденции в востребованности более эффективных инновационных сахароснижающих препаратов, однако до сих пор происходит доминирование ценовой доступности ЛС над целесообразностью их клинического применения, а высокий процент оборота препаратов в коммерческом секторе, вероятно, говорит о недостаточном финансировании лекарственного обеспечения пациентов с СД.

**а:**

а.

## Abstract (en)

BACKGROUND: Procurement of medicines reflects the demand and frequency of prescribing certain drugs, which makes it possible to assess the quality of medical care and compliance with standards. The Russian pharmaceutical market is dynamically developing and expanding, therefore, the commercial sector of drug circulation is a significant part of it and should be studied along with public procurement. Given the significant number of patients diagnosed with diabetes mellitus (DM) in our country, we considered it appropriate and interesting to analyze the structure and volume of turnover of antidiabetic drugs in the retail trade over five years.AIM: to assess the dynamics of the cost and sales volumes of hypoglycemic drugs in the commercial sector for 2019–2020 compared to 2016.MATERIALS AND METHODS: The analysis was made on the basis of the data of antidiabetic drugs purchases in Russian pharmacies in 2016 and 2019–2020, according to 95257 pharmacies data.RESULTS: In 2020, compared to 2016, we see a significant increase in the number of packages purchases (+14,952,897 rub.) and the purchases total amount (+9,377,975,722 rub.), in parallel with the increase in average price per box of the hypoglycemic drug +199, 57 rub. The average price for DPP4 decreased. The cost per pack of metformin remains one of the lowest, second only to glibenclamide and gliclazide. The most expensive drugs include GLP1 group representatives. Insulin purchases have halved, when budget for GLP1 have increased by 10 times, for SGLT2 by 9.5 times, and for DPP4 by 2.1 times. In 2020, metformin gliclazide, a combination of glibencladimide with metformin, glibenclamide and vildgaliptin remain leaders in the number of purchased packages. The purchase leaders in terms of budget share are: metformin, gliclazide, liraglutide, vildagliptin and dapagliflozinCONCLUSION: There are positive trends in the demand for more effective innovative hypoglycemic drugs, however, the affordability of drugs still dominates over the feasibility of their clinical use, and a high percentage of drug turnover in the commercial sector might indicates insufficient funding for drug provision for patients with diabetes mellitus.

## ОБОСНОВАНИЕ

В 2022 г. сахарный диабет 1 и 2 типа (СД1 и СД2) остается глобальной проблемой как в мире, так и в Российской Федерации (РФ) [1]. Общая численность пациентов с СД в РФ, состоящих на диспансерном учете на 01.01.2021 г., по данным Эндокринологического научного центра, составляет 4 799 552 (3,23% населения РФ), из них с СД1 5,5% (265,4 тыс.), с СД2 92,5% (4,43 млн) [2].

Увеличение потребности в сахароснижающих лекарственных средствах (ЛС) и недостаточная эффективность ранее использованных подходов к терапии, особенно при СД2, привели к активным разработкам в области гипогликемических препаратов и большому объему клинических исследований [3]. Вследствие этого за последние годы на мировой фармацевтический рынок вышло значительное количество инновационных сахароснижающих препаратов новых фармакологических групп (агонисты глюкагоноподобного пептида 1 (арГПП1), ингибиторы дипептидилпептидазы 4 (иДПП4), ингибиторы натрий-глюкозного котранспортера 2-го типа (иНГЛТ2). Более того, новые молекулы и торговые наименования продолжают появляться в виде монопрепаратов и различных комбинаций [4]. Новые группы сахароснижающих ЛС показали хороший профиль эффективности и безопасности [5–11].

Таким образом, современная фармакотерапия СД2 включает в себя препараты очень разных групп, лекарственных форм, разной ценовой категории и доступности, имеющие свои особенности действия и сопутствующие эффекты [12]. Все это позволяет врачу подобрать лечение с учетом всех особенностей пациента [13]. Однако на практике доступность наиболее оптимальных ЛС может быть ограничена за счет из высокой стоимости и проблем с льготным обеспечением.

Лекарственное обеспечение в РФ пациентов с СД, как социально значимым заболеванием, финансируется из государственного бюджета. В государственных закупках присутствуют и новые фармакологические группы гипогликемических ЛС [14], однако эта доля не может покрыть потребности всех пациентов, которые могли бы получить преимуществ от их использования. В связи с таким дефицитом многие врачи отдают предпочтение более старым и менее безопасным препаратам [15]. Кроме того, нередко в поликлиниках случаются перебои с поставками лекарств. Все это приводит к тому, что значительная часть больных СД в РФ покупают сахароснижающие препараты в аптеках за свой счет.

Учитывая значительное число пациентов с диагнозом СД в нашей стране, мы сочли целесообразным и интересным проанализировать структуру и объем оборота противодиабетических ЛС в розничной торговле (ритейле) за пять лет, что является ценной клинической информацией о степени внедрения актуальных рекомендаций по лечению СД2.

Закупки ЛС отражают востребованность и частоту назначения тех или иных препаратов, что позволяет оценить качество оказания медицинской помощи и соблюдение стандартов. Российский фармацевтический рынок динамично развивается и расширяется [16], следовательно, коммерческий сектор оборота лекарств является его значительной частью и должен быть исследован наравне с государственными закупками.

## ЦЕЛЬ ИССЛЕДОВАНИЯ

Целью нашей работы было оценить динамику стоимости и объемов реализации сахароснижающих препаратов в коммерческом секторе фармацевтического рынка за 2019–2020 гг. в сравнении с 2016 г. Для достижения этой цели были поставлены следующие задачи: 1) проанализировать объемы закупок ЛС в российские аптеки и цены на ЛС в 2016, 2019, 2020 гг.; 2) выделить лидеров и тенденции в рамках отдельных препаратов или их групп; 3) соотнести с целесообразностью, безопасностью и эффективностью назначений препаратов-лидеров на основе клинических рекомендаций.

## МАТЕРИАЛЫ И МЕТОДЫ

Был проведен анализ данных по закупкам сахароснижающих препаратов в аптечные организации за 2016 и 2019–2020 гг. Данные были собраны в крупных аптечных сетях и несетевых аптеках разных категорий, расположенных в разных субъектах РФ. Данные были собраны и предоставлены аналитическим агентством GSM Group (Россия).

Таким образом, в анализ были включены данные по розничному обороту препаратов, собранные в 95 257 аптеках по всей России. Репрезентативность и сопоставимость данных подтверждаются идентичной методологией сбора в условиях одного аналитического агентства. Срок 5 лет выбран как достаточный для оценки динамики продаж с учетом выхода на рынок большого количества новых препаратов в начале данного периода.

Характеристика источников данных представлена в таблице 1.

**Table:** Таблица 1. Количество аптек, включенных в анализ (n)

Федеральный округ	Всего аптек в субъекте	Аптеки регионального центра	Сетевые аптеки	Не сетевые аптеки
Центральный ФО	28 704	13 810	24 116	4588
Северо-Западный ФО	8365	4679	7451	914
Южный ФО, включая СКФО, исключая Респ. Крым и г. Севастополь	14 714	4154	11 088	3626
Приволжский ФО	19 432	7239	17 279	2153
Уральский ФО	7481	2366	6713	768
Сибирский ФО	12 038	5175	9858	2180
Дальневосточный ФО	3099	1328	2184	913
Крымский ФО	1424	532	1215	209
Итого	95 257	39 283	79 904	15 351

## Дизайн исследования

Поперечное аналитическое исследование.

## Методы

В анализ включались данные: количество закупленных препаратов (в уп.), стоимость закупленных препаратов (руб.), средневзвешенные цены на отдельные препараты (руб.). Анализ проводили по группам ЛС.

## Статистический анализ

Статистическая обработка материала выполнена в программе Statistica 10 c использованием методов описательной статистики. Дополнительный анализ для оценки статистической значимости качественных различий оценивали по критерию χ² Пирсона с использованием программного обеспечения GraphPad Prizm 5.0.

## РЕЗУЛЬТАТЫ

В ходе анализа данных закупок было отмечено, что в 2020 го., в сравнении с 2016-м, наблюдался значимый рост закупок в количестве упаковок (+14 952 897 шт.) и общей сумме закупок (+9 377 975 722 руб.) на фоне увеличения средневзвешенной цены на одну коробку препарата на 199,57 руб. (табл. 2, рис. 1).

**Table:** Таблица 2. Сводная таблица оборота гипогликемических ЛС в 2016, 2019, 2020 гг.

Годы	Количество упаковок,шт.	Общая сумма закупок, руб.	Средневзвешенная цена, руб.
Всего
2016	31 327 911	11 432 760 898	840,71
2019	46 802 240	18 883 933 891	981,81
2020	46 280 808	20 810 736 620	1040,24
Инсулины
2016	2 597 185	2 990 360 329	1 236,12
2019	2 318 120	1 475 028 537	1 429,15
2020	1 853 152	1 310 444 931	1 379,83
иДПП4 (глиптины)
2016	1 720 268	1 939 041 730	1 774,25
2019	3 320 020	3 911 285 055	1 638,68
2020	3 759 184	4 195 392 863	1 555,87
Аналоги арГПП1
2016	27 07	216 240 980	5 812,75
2019	141 54	1 631 881 157	9 857,53
2020	189 489	2 186 905 964	9 999,73
иНГЛТ2 (глифлозины)
2016	115 841	242 661 092	2 295,6
2019	696 677	1 785 837 983	2 643,37
2020	956 233	2 325 974 887	2 790,87
Сульфонилмочевина (в т.ч. комбинированные)
2016	14 081 926	3 260 485 450	312,98
2019	17 094 214	4 597 203 541	319,53
2020	16 426 809	4 480 556 530	394,42
Метформин (монопрепараты)
2016	12 759 729	2 751 722 473	219,69
2019	23 004 292	5 392 682 711	184,77
2020	22 966 360	6 255 294 521	252,31

**Figure:**
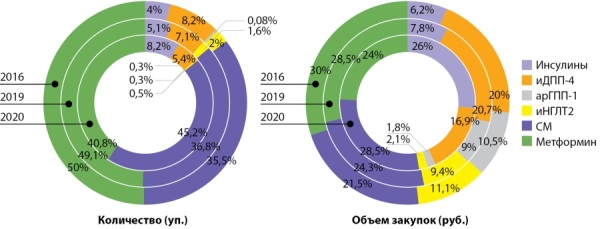
Рисунок 1. Распределение объема закупок различных групп сахароснижающих препаратов в 2016, 2019, 2020 гг.

Возросли закупки всех групп ЛС, кроме инсулинов, чей оборот снизился в два раза (- 744 033 шт. и -1 679 915 398 руб.). Из важных тенденций стоит отметить снижение средневзвешенной цены на ЛС группы иДПП4 с 2016 года. на 218,38 руб. (табл. 2).

С помощью критерия χ² выявлено, что динамика распределения групп препаратов с 2016 по 2020 гг. различалась с высокой статистической значимостью (р<0,0001), что свидетельствует не только о количественном росте продаж и/или стоимости отдельных ЛС, но также о наличии качественных изменений в структуре потребления.

В 2020 г. абсолютными лидерами по объему реализации остаются монопрепараты метформина и группа сульфонилмочевины (СМ) как по количеству упаковок (35,5 и 21,5% соответственно), так и по общей сумме закупок (49,8 и 30% соответственно).

Стоит отметить, что в 2020 г. закупки препаратов группы иДПП4 (в рублях) достигли 20,1% и почти сравнялись долей с препаратами СМ (21,5%) (рис. 1).

Средняя стоимость упаковки метформина остается одной из самых низких (217,69 руб.), уступая только глибенкламиду и гликлазиду. К самым дорогим препаратам относятся все арГПП1, средняя цена в группе составляет 11 333,79 руб. (рис. 2).

**Figure:**
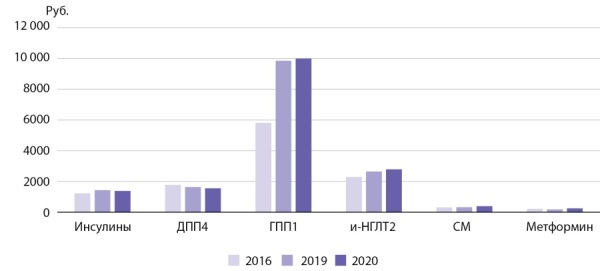
Рисунок 2. Средневзвешенная цена на 1 упаковкусахароснижающих лекарственных средств.

Кроме монопрепаратов метформина, лидерами закупок по количеству являются гликлазид, комбинация глибенкламида с метформином, глибенкламид и вилдаглиптин. Лидерами закупок по доле в бюджете являются метформин, гликлазид, лираглутид, вилдаглиптин и дапаглифлозин (рис. 1, табл. 1).

Группа препаратов иДПП4 отличается благоприятным профилем сердечно-сосудистой безопасности, не приводит к развитию гипогликемий, в том числе ночных и, следовательно, не вызывает увеличения массы тела пациентов. Указанные преимущества обуславливают возрастающую востребованность данных ЛС и увеличение их доли в обороте ПССП. иДПП4 характеризуются средней активностью в снижении уровня гликированного гемоглобина (HbA1c) и хорошим сроком удержания эффекта [5]. Ряд публикаций свидетельствует также о протективном действии на бета-клетки поджелудочной железы [6], что представляется крайне перспективным.

Вилдаглиптин остается наиболее востребованным ЛС из иДПП4 в 2020 г. — как в виде монопрепарата (Галвус), так и в комбинации с метформином (ГалвусМет). Значимую долю представляют алоглиптин (Випидия) и ситаглиптин (Янувия, Кселевия), в т.ч. комбинации с метформином (табл. 3, рис. 3).

**Table:** Таблица 3. Оборот препаратов группы иДПП4 в 2016, 2019, 2020 гг.

Препарат	Год	Количество упаковок, шт.	Общая сумма закупок, руб.
Алоглиптин	2016	65 559	74 152 682
2019	390 745	486 233 113
2020	430 181	536 207 429
Вилдаглиптин	2016	1 442 196	1 458 548 537
2019	2 408 846	2 410 509 276
2020	2 690 919	2 482 315 973
Линаглиптин	2016	48 326	73 177 016
2019	140 976	235 270 045
2020	174 160	285 991 689
Ситаглиптин	2016	131 221	262 044 313
2019	327 040	663 286 486
2020	398 834	769 699 600
Саксаглиптин	2016	28 966	72 119 182
2019	51 601	115 386 223
2020	51 937	111 197 058
Гозоглиптин	2016	0	0
2019	812	599 912
2020	11 675	8 697 007
Эвоглиптин	2016	0	0
2019	0	0
2020	1 478	1 284 107

**Figure:**
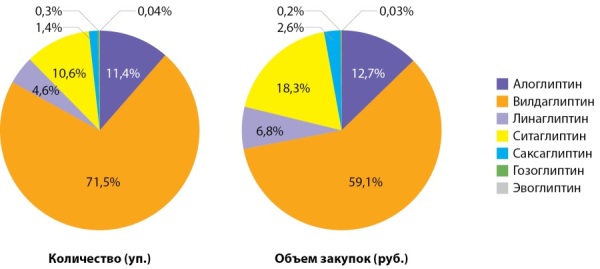
Рисунок 3. Распределение количества упаковок и объема закупок иДПП4 в аптечных организациях, 2020 г.

Обращает на себя внимание неожиданно низкий оборот препаратов саксаглиптина (Онглиза, Комбоглиз) в коммерческом секторе, тогда как именно эти препараты превалируют в государственных закупках и составляют большую часть назначений эндокринологов в городских поликлиниках.

ЛС из группы арГПП1 являются инновационными препаратами, которые эффективно и мощно влияют на патогенез СД2 на нескольких уровнях. Данная группа препаратов не только восстанавливает физиологическую реакцию инсулина на прием углеводов и нормализует обмен глюкозы, но и выраженно снижает массу тела пациентов [7][8]. Лидерство в группе арГПП1, как и в 2016 г., остается за лираглутидом, который представлен на рынке в виде двух форм — Саксенда и Виктоза (54,6% доля в рознице, 63,1% доля в бюджете). Отмечается при этом снижение реализации препарата на фоне выхода на рынок дулаглутида (Трулисити) и семаглутида (Оземпик), ликсисенатида в комбинации с инсулином гларгин (Соликва) (рис. 4, табл. 4).

**Table:** Таблица 4. Оборот группы препаратов арГПП1 в 2016, 2019, 2020 гг.

Препарат	Год	Количество упаковок, шт.	Общая сумма закупок, руб.
Эксенатид	2016	7 110	31 830 279
2019	2 786	15 086 904
2020	827	5 328 760
Лираглутид	2016	19 454	182 375 605
2019	92 727	1 136 114 262
2020	103 564	1 380 187 206
Ликсисенатид	2016	507	2 035 096
2019	3 629	16 871 339
2020	19 513	24 478 362
Семаглутид	2016	0	0
2019	0	0
2020	12 898	126 004 765
Дулаглутид	2016	0	0
2019	42 399	463 808 652
2020	64 258	635 287 311

**Figure:**
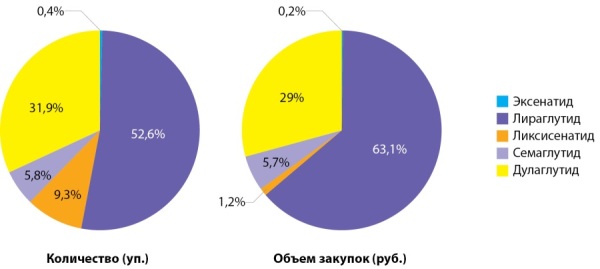
Рисунок 4. Распределение количества упаковок и объема закупок арГПП1 в аптечных организациях, 2020 г.

Рост закупок препаратов группы иНГЛТ2 в 9,5 раза с 2016 г. можно интерпретировать как позитивную тенденцию в связи с доказанными во многих международных исследованиях эффектами кардио- и нефропротекции этих ЛС [9][10][17]. В 2020 г. первенство в группе поровну делят дапаглифлозин (Форсига) и эмпаглифлозин (Джардинс), в том числе в комбинации с метформином. Средневзвешенная цена дапаглифлозина при этом остается несколько ниже средневзвешенной цены эмпаглифлозина (табл. 5, рис. 5).

**Table:** Таблица 5. Оборот группы препаратов иНГЛТ 2 в 2016, 2019, 2020 гг.

Препарат	Год	Количество упаковок, шт.	Общая сумма закупок, руб.
Дапаглифлозин	2016	90 238	194 949 104
2019	377 074	881 805 851
2020	504 094	1 110 579 917
Канаглифлозин	2016	899	2 693 752
2019	7450	26 526 430
2020	6329	22 098 860
Эмпаглифлозин	2016	24 704	45 018 236
2019	311 255	874 721 936
2020	426 937	1 137 746 487
Ипраглифлозин	2016	0	0
2019	898	2 783 766
2020	18 873	55 549 623

**Figure:**
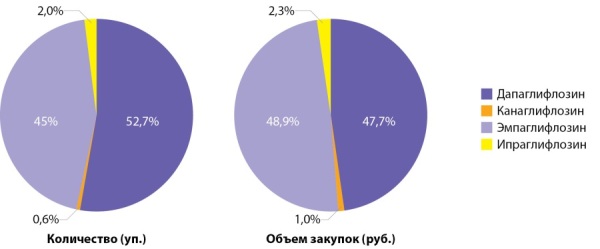
Рисунок 5. Распределение объема упаковок и закупок иНГЛТ2 в аптечных организациях, 2020 г.

Группа препаратов СМ хорошо изучена и давно применяется в терапии СД2. Эти ЛС достаточно снижают уровень HbA1c, и долгое время были во 2-й линии терапии при недостаточной эффективности метформина в отношении снижения уровня HbA1c. Однако именно эта группа сопряжена с низким профилем безопасности, прежде всего — высоким риском гипогликемий, приводящих к последующему увеличению потребности в пище и гипергликемии [3].

Есть данные, что препараты СМ своим мощным влиянием на секрецию инсулина приводят к быстрому истощению аппарата бета-клеток поджелудочной железы, снижению контроля терапии, повышению потребности в инсулине и быстрому развитию осложнений [15].

Гликлазид и комбинация глибенкламида с метформином лидируют в группе препаратов СМ. Меньший процент приходится на глимепирид. Такой препарат группы СМ, как глипизид, исчез из аптечных закупок в 2019–2020 гг. (рис. 6, табл. 6).

**Table:** Таблица 6. Оборот группы препаратов сульфонилмочевины в 2016, 2019, 2020 гг.

Препарат	Год	Количество упаковок, шт.	Общая сумма закупок, руб.
Глибенкламид	2016	2 048 121	247 624 688
2019	2 170 762	295 872 506
2020	1 838 829	258 728 110
Глибенкламид+метформин	2016	3 427 320	804 845 575
2019	3 154 517	920 382 341
2020	2 970 049	944 308 175
Гликвидон	2016	146 489	53 741 983
2019	140 545	64 403 460
2020	131 883	62 071 523
Гликлазид	2016	7 135 277	1 504 051 996
2019	10 096 354	2 423 716 714
2020	10 057 802	2 322 969 706
Гликлазид+метформин	2016	51 359	16 620 370
2019	52 386	12 294 610
2020	27 026	12 284 672
Глимепирид	2016	1 060 706	517 524 472
2019	1 291 887	724 696 689
2020	1 197 788	708 697 986
Глимепирид+метформин	2016	212 217	116 014 831
2019	187 763	155 927 194
2020	203 432	171 496 358
Глипизид	2016	437	61 535
2019	0	0
2020	0	0

**Figure:**
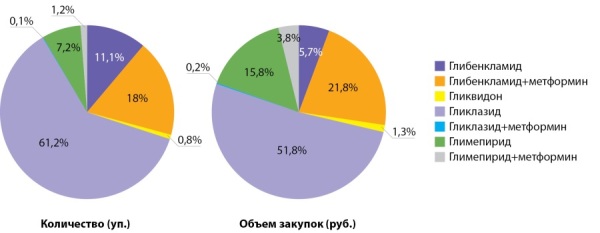
Рисунок 6. Распределение количества упаковок и объема закупок ЛС из группы СМ в аптечных организациях, 2020 г.

## ОБСУЖДЕНИЕ

Период 2019–2020 гг. отражает картину максимального количества продаж инновационных ЛС терапии СД2, вышедших на рынок в 2016–2019 гг. За счет большого количества разносторонних клинических исследований и активной работы фармацевтических компаний в отношении медицинской информации, несмотря на недолгий период введения в практику, новые ЛС активно используются и очевидно вносят позитивный вклад в компенсацию и клинические исходы СД2. Например, с 2016 г. до 2020 г. средняя длительность СД2 до момента смерти пациентов увеличилась с 11,0 до 11,4 года. Доля пациентов с целевым HbA1c<7% повысилась с 51,9 до 52,1%, с HbA1c≥9,0% — уменьшилась с 8,9 до 8,0% [2].

Согласно полученным данным, можно говорить о нескольких позитивных тенденциях в структуре закупок сахароснижающих препаратов.

Особый интерес представляет снижение оборота инсулинов в 2 раза в коммерческом секторе с 2016 г. по 2020 г. — как по количеству упаковок, так и по доле в бюджете (в процентах и в абсолютных цифрах). Данная тенденция, по мнению авторов, связана с улучшением в этот период льготного обеспечения препаратами инулинов.

По данным агентства Headway Company, в январе-июне 2020 г. объем государственных закупок препаратов достиг 164 млрд руб., что на 41% больше, чем за тот же период 2019 г. В частности, «Герофарм» в 2020 г. удвоил продажи в госсекторе, обеспечив более 10% закупок, а объем госконтрактов компании на инсулины в 2020 г. вырос более чем вдвое, до 1,8 млрд руб. Основную долю составили закупки аналогов лантуса и хумалога. При этом в 2022 г. на фоне большого количества отмененных тендеров и снижения объемов льготного обеспечения снова наблюдается рост продаж инсулинов в аптеках (данные Headway Company). Таким образом, можно говорить о зависимости оборота препаратов в коммерческом секторе от степени государственного обеспечения.

Тем не менее высокий процент оборота препаратов в коммерческом секторе говорит о все еще недостаточном финансировании лекарственного обеспечения пациентов с СД2 за счет государства, что в первую очередь касается препаратов с неистекшим сроком патента и не имеющих генерических аналогов.

Анализ предоставленных нами данных во многом соответствует опубликованным данным официального регистра, составленного на основании назначений государственных поликлиник [2]. На 2020 г. структура терапии при СД2: пероральные препараты (ПССП) — 76,2% пациентов (монотерапия — 44,1%; комбинация 2 ПССП — 28,9%, 3 ПССП — 3,2%), инсулинотерапия — 18,8%, без медикаментозной терапии — 4,9%. Сократилось количество пациентов на монотерапии инсулинами (с 8,3 до 7,9%) и получающих один ПССП (с 51,5 до 46,4%), возросло количество назначений 2,3 и более ПССП. В качестве монотерапии и комбинации 2 препаратов со значительным перевесом преобладают метформин и СМ (до 82% назначений). Однако доля иДПП4 выросла с 3,5 до 8,95%, иНГЛТ2 — c 0,5 до 3,3%, арГПП1 — с 0,1 до 0,3% [2].

Таким образом, среди лидирующих препаратов и в государственном, и в коммерческом секторе в первую очередь необходимо отметить относящиеся к ценовому сегменту до 500 руб. за упаковку. Именно средства, стоящие меньше этой суммы, составляют основу фармацевтического рынка сахароснижающих препаратов, к ним относятся препараты производные СМ и метформин.

Инсулин и его аналоги также занимают значительное место как в государственном, так и в коммерческом сегменте рынка, а востребованность инновационных препаратов оказывается значительно выше в коммерческом секторе. Все это свидетельствует о вероятной нехватке льготного обеспечения этими препаратами.

Данная информация отражает ситуацию с реализацией ЛС терапии СД2 по продажам, но не по назначениям, при этом тенденция сохраняется. Однако можно утверждать, что значительную часть рынка сахароснижающих средств занимает коммерческий сектор. Исходя из представленных данных, можно сделать вывод о востребованности новых и более эффективных ЛС, а также необходимости их внедрения на фармацевтический рынок РФ, при этом цена реализации остается значительной проблемой, ограничивая доступность большей части граждан к инновационным препаратам.

## ОГРАНИЧЕНИЯ ИССЛЕДОВАНИЯ

Представленные нами данные отражают значительную долю объема ритейла, но представляют только часть оборота гипогликемических средств в РФ. Данные по закупкам ЛС динамические, в связи с этим сложны в сборе и интерпретации, поэтому могут быть неоднородными. Наше исследование позволяет только отразить ряд тенденций.

## ЗАКЛЮЧЕНИЕ

## ДОПОЛНИТЕЛЬНАЯ ИНФОРМАЦИЯ

Источники финансирования. Работа поддержана РНФ (проект №20-75-10013).

Конфликт интересов. Авторы заявляют об отсутствии конфликта интересов.

Участие авторов. Куркин Д.В., Колосов Ю.А. — разработка концепции исследования и научное консультирование; Бакулин Д.А., Робертус А.И., Иванова О.В., Кудрин Р.А. — сбор материала, составление таблиц; Крысанов И.С. — статистический анализ; Макарова Е.В. — написание текста статьи.

Все авторы одобрили финальную версию статьи перед публикацией, выразили согласие нести ответственность за все аспекты работы, подразумевающую надлежащее изучение и решение вопросов, связанных с точностью или добросовестностью любой части работы.
